# A Phone-Based Early Psychological Intervention for Supporting Bereaved Families in the Time of COVID-19

**DOI:** 10.3389/fpubh.2021.625691

**Published:** 2021-02-02

**Authors:** Lidia Borghi, Julia Menichetti, Elena Vegni, The Early Bereavement Psychological Intervention working group

**Affiliations:** ^1^Clinical Psychology, Department of Health Sciences, University of Milan, Milan, Italy; ^2^Institute of Clinical Medicine, Akershus Universitetssykehus, University of Oslo, Oslo, Norway; ^3^Unit of Clinical Psychology, Santi Paolo and Carlo Hospital, Milan, Italy

**Keywords:** bereavement, clinical psychology, COVID-19, family, hospital psychology, phone follow-up care, preventive primary intervention, psychological intervention

## Abstract

The exceptional circumstances of the Coronavirus disease (COVID-19) pandemic are making the grief processes challenging for families who are losing a relative for COVID-19. This community case study aimed to describe a phone-based primary preventive psychological intervention that has been delivered to these families by the Clinical Psychology unit of an Italian hospital. In particular, the article reports how the intervention has been organized within the overall hospital care pathway for families, the specific contents and components of the intervention, and the seven-phase structure of the intervention. The unique features and related challenges of the intervention, along with the implications for clinical practice, are discussed.

## Introduction

The Coronavirus disease-19 (COVID-19) pandemic has been generating a global health crisis, with overall death rates surpassing 1.1 million people worldwide and continuously increasing, as of 28th October 2020 (1). The threats of the pandemic to the health of the worldwide population, the safety measures that require physical distancing, and the rate of the contagion that burdens the healthcare systems have been creating extraordinary, extremely challenging circumstances for how people affected by COVID-19 die and how families can come to terms with the loss.

Experiencing a loss, and unfortunately in this pandemic sometimes also multiple losses, in a condition of isolation can be extremely difficult for family members. The last goodbye, the closeness and support of the family and of the social group, and the funeral rituals have always been a crucial part of the process of realizing and coming to terms with the loss of a loved one. All these aspects have built, over time, the metaphorical road that allows processing and separating from the deceased, and that allows moving through grief, loss and transition. The COVID-19 pandemic can affect some of these basilar stones that enable and support the grieving process. The physical distancing can, indeed, limit the possibility to receive social support. Moreover, especially in the emergency phases of the pandemic, the regular grieving rituals have been frequently limited or even banned. For example, during the emergency phase of the first pandemic wave in Italy, all funeral ceremonies were banned for about 2 months, restricting family members from seeing the body of the deceased, from attending the cremation or the burial, or from going to the cemetery. Especially for families who have experienced a COVID-19 related loss in the hospital, the mourning can be particularly at risk, as there may have been no one in the family who has been able to testify the last days of life of the loved one and his/her death. Furthermore, such deaths are often quick and unexpected, as hospitalized patients are those most severely affected by the virus. The unexpectedness of the loss has been proved to explain stronger grief reactions among family members who have lived a COVID-19 related loss than among those who have lived a natural loss, similarly to what happens after unnatural losses (2) Finally, the families experiencing a loss in an intensive care unit can particularly be affected by the loss: end of life care can proceed fast and this can challenge the possibility to obtain timely, consistent, and clear information (3).

For all these reasons, bereaved families dealing with a COVID-related loss at the hospital can be particularly in need of support from the hospital team. A recent study from our group (4) has pointed out the nature of these needs: to give meaning to the lived experience; to express emotions; to say the last goodbye; to remember the loved one; and to solve practical issues. However, it is unclear how hospitals should reorganize their services to meet these multiple needs, together with the challenge of managing COVID-19 patients and providing at the same time the usual care to all the other patients.

Supporting families of patients who are dying and died in the hospital should be an on-going task of the healthcare system, going beyond the care provided to the patient and continuing also during the days after the loss (5, 6). However, the scarcity of time, the lack of resources, and the safety restrictions, which especially featured the emergency phases of the pandemic, can limit the possibility to support bereaved families (7).

During the emergency phases of the COVID-19 pandemic, and especially in the most affected areas, hospitals had to reorganize their services and healthcare professionals' tasks to face the COVID-19 related challenges (i.e., a large number of patients who needed hospitalization and intensive care treatments, high rate of simultaneous deaths). In these periods, several clinicians had to reorient their professionality to address the emergent needs of the pandemic (e.g., the non-essential care like routine follow-ups was limited, some clinicians working in other units had to move to COVID-19 units, clinicians with specific vulnerabilities for COVID-19 were limited from direct patient care, or, in some countries, retired doctors were invited to volunteer in the hospitals) (8). This reorganization involved also the clinical psychology units. For example, in China, some authors during the beginning of the COVID-19 outbreak claimed the need for prompt mental health care for people affected by COVID-19 and suggested to implement online services, as non-essential healthcare personnel such as psychologists, psychiatrists, and mental health workers were often limited from providing direct patient care and/or from accessing isolation wards or rooms for patients affected by COVID-19 (9). Other authors followed this suggestion by proposing, even if without evidence of their effectiveness for COVID-19 patients and families, the use of eHealth/remote care (e.g., telephones or internet platforms) as a feasible way for psychologists to deal with the urgent psychological challenges related to the pandemic and to provide psychological support to patients, families, and the medical staff (10–12). In Lombardy, Italy, the activities of the clinical psychologists were maintained and delivered also to hospitalized patients with COVID-19 (13), and, when possible, they were provided remotely. Moreover, recent contributes have proposed literature-based recommendations for specifically supporting COVID-19 bereaved families and preventing dysfunctional grief (14–16). Among the many recommendations, all these contributions suggested (but without empirical evidence supporting the claim) the importance of phone follow-up to families after the loss, as a mean to assess more serious symptoms that may require additional support.

However, to our knowledge, there are no studies describing in detail psychological interventions that have been delivered to specifically address the early needs of bereaved families who lost a loved one for COVID-19 at the hospital.

The purpose of the present community case study is to describe the local experience of the Clinical Psychology Unit of a large public healthcare organization in Milan (Lombardy, Italy) in delivering a phone-based early psychological intervention to families of hospitalized patients who died for COVID-19 during the first-wave of the pandemic. In particular, we describe how the intervention is organized within the hospital care pathway for families, the specific contents, components and structure of the intervention, and discuss the implications for clinical practice and for further research studies.

## Context

The Azienda Socio Sanitaria Territoriale (ASST) Santi Paolo and Carlo is a large public healthcare organization in Milan covering hospital and community care services (it provides more than 150,000 emergency services/year, with a daily flow of 40/50 emergency patients). It is composed of two main hospital facilities connected to the University of Milan. A clinical psychology unit within the hospital offers psychological assessment, psychological support and psychotherapy to adults with psycho-emotional or psychopathological sufferance related to medical conditions (17). The psychologists' theoretical models of reference are various (e.g., cognitive-behavioral, psychodynamic, systemic, person-centered) but they all share an expertise in providing psychological support to hospitalized patients (and their families) affected by a medical condition.

Since the beginning of March, when the pandemic started spreading consistently in Milan, and particularly during the emergency phases of the pandemic, the clinical psychology unit of the ASST reoriented part of its activities to address the emerging psychological needs of the hospital caused by the COVID-19 pandemic. This reorganization has been structured by a constant dialogue with the hospital managers, clinicians, patients and their families. One of the first areas in need of a psychological intervention emerged by this dialogue has been the management of the COVID-19 deaths, for its two-fold impact on the hospital care pathway and on families' support needs. Indeed, clinicians, patients, and their families were all in urgent need of managing the emotional difficulties of experiencing a high number of simultaneous deaths and of living the safety measures in place against the virus.

To face this double need of early assessing and supporting bereaved families and lightening the workload of clinicians, the clinical psychology unit decided to deliver a phone-based early psychological intervention to all the families of COVID-19 deceased patients, about 48–72 h after the family received notification of the death of the loved one from the hospital clinician. In the period between March 19th and June 15th, 284 families were called and 246 family members received the intervention (38 family members were unreachable). From what the psychologists performing the calls reported in their written reports after each call, the majority of family members felt grateful for the call and for the support.

## Overview

### The Hospital Care Process for Bereaved Families of COVID-19 Victims

**Figure 1:**
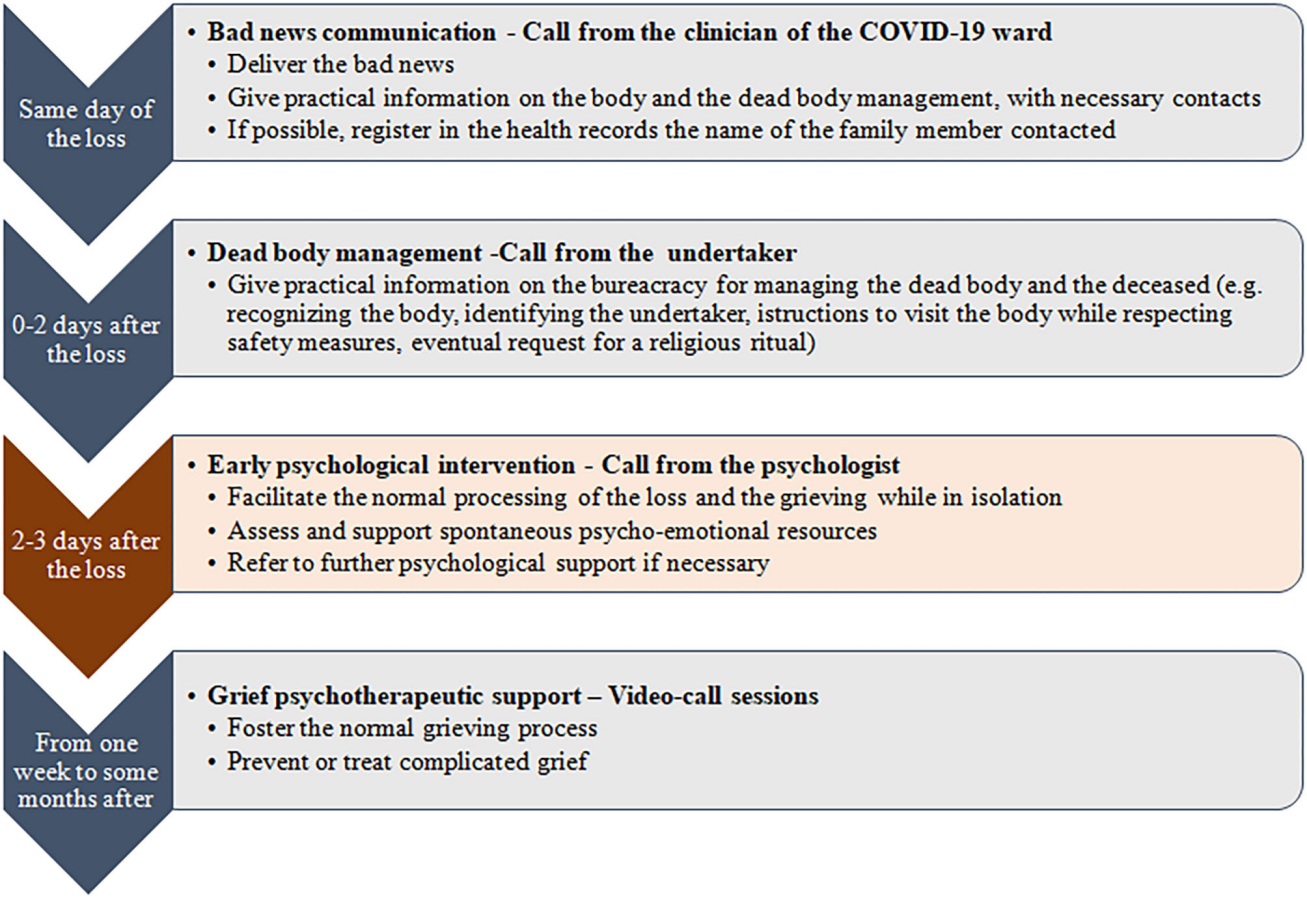
The process of care to families of COVID-19 deceased patients at the hospital.

The phone-based early psychological intervention was part of a multidisciplinary and integrated-care process to support the families who lived the loss of a loved one for COVID-19. Indeed, the phone call served as the closure of the hospital care pathway and as a link, eventually, to other community-based support services. Therefore, it may be positioned as a primary preventive intervention within a stepped care model, where different professionals were involved in different bereavement care phases: (1) the communication of the death, (2) the management of practical aspects related to the death, (3) the primary preventive phone-based psychological intervention, and, eventually, (4) the community-based psychotherapeutic intervention. The psychologists delivering it were not involved in the support of the dying patients affected by COVID-19. Figure 1 exemplifies the process of care to the families who lived the loss of a family member for COVID-19 at the hospital.

As a first step, the clinician who was taking care of the patient called the designated family member (meaning the family member chosen by the patient to receive communication about his/her care and health status) to communicate the bad news. The family member also received contact from the undertaker to dispose the burial, manage the dead body, and formalize the death. Then, the hospital management notified the Clinical Psychology Unit of the ASST of the death. A group of 14 clinical psychologists of the ASST with an expertise in conducting psychological consultations with hospitalized patients and their families (e.g., managing the emotional or behavioral reactions to a diagnosis, to treatment options, or to an unfavorable prognosis, or to end-of-life care) performed the phone follow-ups to families. In detail, after 2–3 days from the communication of the loss and after having checked with the mortuary that the previous steps were accomplished, the clinical psychologists obtained information on the designated relative from the electronic health records and called the family member. The designated family member was the relative previously selected by the patient to receive notifications by the hospital staff, and she/he usually was the closest person to the patient. When the psychologists made the call, they asked about the relationship with the deceased (e.g., partner, son/daughter, brother/sister, and nephew) and about other relatives in need of psychological support. In this case, they made another phone call to the indicated relative. In average, the call lasted 30–40 min (with a range from 10 to 60 min). Such call served as a psychological intervention to support all families, and in particular to foster and sustain spontaneous strategies and resources, but also to assess psycho-emotional difficulties and risk factors that may have required further specialized support. For example, the psychologists assessed, during an open dialogue with the family member and/or with focused screening questions, the relative's emotional response to the loss, the previous presence of physical and/or psychiatric disorders or other personality traits predisposing to psychological vulnerability, the coping strategies and defense mechanisms in play, and the availability of social resources. After this assessment, the psychologist offered to the family member who was evaluated to be at risk for grieving difficulties the possibility to be referred to further psychological support by a team of community-based psychologists. Such a team provided individual psychotherapeutic grief sessions by video-calls, with a flexible duration based on the family member's needs and recovery trajectory.

### Unique Features of the Phone-Based Early Psychological Intervention

Several aspects characterized the psychological intervention offered by the Clinical Psychology unit to all families of hospitalized patients who died for COVID-19, and made it unique (and unusual for psychologists): (a) no prior referral or self-referral, (b) multiple purposes, (c) critical timing (i.e., a single conversation in proximity of the loss) (d) lack of face-to-face contact, and (e) virtual setting.

First, as the call was part of the hospital care and was aimed to intercept eventual needs for information and/or further support, the intervention was delivered to all families of the deceased patients. In delivering the call, the psychologists “knocked at the patients' door,” rather than the opposite. This is uncommon for psychological interventions, where usually the patient is self-referred or referred by others and is willing to contact a psychologist. This was challenging for psychologists and activated feelings of anxiety, worry, invasiveness, doubt, or unsafety. A useful strategy adopted by the psychologists to manage these feelings was to find verbal formulas to show caution, to clarify the purpose of the contact from the very beginning, and to be open to accept a wide range of reactions from the family member.

Second, apart from the previously explained relief of clinicians, this intervention covered multiple purposes, which changed from call to call depending on the family member's needs that emerged at each call. For example, some calls mostly had the function of collecting and solving doubts related to practical/procedural/bureaucratic issues; other calls worked as a space for the relative/family to express the loss-related emotions, facilitating grief reactions while in physical isolation or quarantine; other calls again mostly verified and sustained spontaneous psycho-emotional resources, by facilitating alternative death rituals and reassuring the family member that the loved one was not alone in the process of death; finally, in some cases, if the psychologist observed highly-complex/at-risk situations, the calls had the function to refer the family member to further psychological support.

Then, the intervention was delivered in a specific timeframe: 48–72 h after the death notification. This timeframe was chosen to allow the family to deal with the preliminary logistic aspects of the burial and to have time to emotionally connect with the loss. The same timeframe, quite close to the loss, would have also allowed to early intercept relatives in need and to cover the previously described purposes. This is an unusual timeframe for psychologists for providing psychological support to families. Indeed, such a follow-up is usually delivered by healthcare professionals, and psychologists take care of the grief support that eventually follows after it.

Moreover, the intervention had to be delivered by phone due to safety restrictions. Psychologists could not rely on the family member's facial expressions, gestures, and use of space to fine tune their action, nor on their own ones to fully convey their messages. The tone of voice, both of the family member and of the psychologist, was invested by an extremely important role to collect and provide all the additional information usually conveyed by the body when in physical presence. The same modality also affected the setting, which was unclear and aleatory. The psychologists were thus deprived of an important resource/tool. At the same time, hospital psychologists are used to deal with unusual settings (e.g., when providing psychological support at the patient's bed). The psychologists involved in the calls had particularly to find ways to re-create a psychological setting and a psychological role in their mind, and to find the “right distance” from the relative. Indeed, they felt both too distant (e.g., because of the phone, because they did not know and were not able to see the respondent, because they acted as part of the hospital care, because they had only one single conversation with the family member) and too close to the relative (e.g., because they were involved in the COVID-19 situation themselves, because they were “knocking at the other's door”). The psychologists used different strategies to empathize/go closer to the respondent (e.g., by paying attention on the tone of voice, rhythm, and warmness in their voice, by slowing the speaking rate) or to take distance (e.g., by referring to other resources, by stressing the purpose of the call, by introducing some pauses in dialogue).

### A Structured Model of the Intervention

Before delivering the intervention, it was decided that the clinical psychologist performing the call had to present him/herself as psychologist and member of the hospital and that she/he had to clearly state that the call was part of the hospital care. Thus, during the setup of the intervention, a brief speech outline was shared among the clinical psychologists: introducing him/herself with name and role (i.e., psychologist employed in the hospital); asking for a confirmation of the interlocutor's identity as the designated relative of the deceased patient; presenting the call as part of the hospital care; and asking the consent to proceed in the talk. This preliminary intervention structure was refined during the clinical practice with 246 families and based on families' emerging needs (4). As Table 1 resumes, the final result was a structured intervention model, with seven main phases each with specific objectives, strategies and verbal and para-verbal communication skills.

**Table 1:** The structure of the phone-based psychological intervention for COVID-19 bereaved families.

**Phases**	**Objectives and strategies**	**Skills and techniques**
Opening	Check the respondent's identity, introduction with name and role (i.e., psychologist employed in the hospital), consent to proceed	Accurate presentation Use of verbal formulas to show caution Attention to the respondent's tone of voice to fine tune the intervention Question to check the willingness of the relative to proceed in the call
Proactive offer	Reason of the call: offer a free space to talk	Clear focus on the reason of the call Use of pauses
Active listening	Active listening of family member experiences, thoughts and emotions	Attentive silence Use of para-verbal signals to facilitate the spontaneous communication flow
Assessment	Assessing psycho-emotional needs, psycho-social resources and risk factors	Open- and close-ended focused screening questions
Need-based psychological actions	Information giving, education on stages of grief, emotional validation, small therapeutic actions like cognitive reframing and relaxation pills	Various, ranging from portioning, organizing and prioritizing education and information based on respondent's needs to supportive statements and reframing arguments under new angles
Referral and connection	Indications about the resources offered by the hospital and the community-based services, eventual referral to further psychological support	Give information in small bits Check the family member's understanding
Closure	Say goodbye	Use of tone of voice and verbal formulas to emphasize closeness/warmness

In particular, for the sixth phase (referring to further psychological support), the psychologists based their evaluation on a punctual assessment of very early protective and risk factors that might have facilitated the development of complicated grief in the future. The main very early risk and protective factors assessed by the psychologists are summarized in Table 2, which were based on literature evidence about generic risk/protective factors for dysfunctional grief (18, 19) and the experience of clinical psychologists with each family member contacted in the first days after the loss. In particular, the assessment of risk and protective factors was focused not only on checking the presence/absence of well-known evidence-based factors affecting the grief, but also on eliciting and sustaining spontaneous coping strategies and resources of family members (see also Borghi and Menichetti, under review), and, finally, evaluating how much the complex interplay of very early risk and protective factors may have potentially affected the normal bereavement process in each family member.

**Table 2:** Very early risk and protective factors assessed by psychologists during the call.

**Risk factors**	**Protective factors**
* Individual factors (personality traits, psychiatric history, and previous traumas) * Type of death (e.g., rapid, unexpected, and untimely) * Death in the intensive care unit * Uncertainty, lack of information, and poor communication with the hospital staff * Lack of emotional and social support due to lack of social networking and/or social distancing * Physical distancing (not having had the chance to stay with the relative in the last period of life and to say the last goodbye)	* Resilience * Creativity and flexibility: to be able to find new ways to cope and to adapt to the grief under the extraordinary circumstances of COVID-19 * Gratefulness and good communication with the hospital staff * Faith, spirituality, and religious beliefs

## Discussion

The existing literature on grief therapy is mostly focused on how to provide psychological support to treat complicated grief (18) or how to support bereaved persons, for example through the reconstruction of meaning (20). Differently, the phone-based early psychological intervention that emerged from the hospital work with COVID-19 patients and bereaved families appears to be more close, due to its timing, functions, and format, to a bereavement follow-up as part of the hospital care. Bereavement follow-ups are usually provided by nurses, volunteers, social workers, and the type of support can range from giving a booklet or condolence letter to providing individual/group support (21). In this case, it was structured as a brief psychologist-led intervention due to the complexity of grieving during COVID-19 and to the limited hospital resources during the emergency. It also presented characteristics similar to the early psychological interventions that are usually provided in situations of emergency, which usually provide prompt assessment of individuals at risk, debriefing and promotion of coping skills and resilience (22). Furthermore, the intervention was aligned with recent literature-based recommendations for supporting COVID-19 bereaved families and preventing dysfunctional grief (14–16). Among the many recommendations, these studies highlighted the importance of an organized action with multidisciplinary healthcare staff, of using e-devices and telephone above the others, and of providing early follow-ups after the loss as a mean to assess more serious symptoms that may require additional support. Therefore, the phone-based early psychological intervention that has been delivered to address the specific needs of bereaved families who lost a loved one for COVID-19 at the hospital can be conceived as at an interface between bereavement follow-ups and emergency psychological interventions, aligned with emerging recommendations for specifically supporting this target group. Even if it requires further evaluation, it might represent a helpful and feasible support for families to cope with the very early moments of the bereavement process and prevent further distress and risks of complicated grief. Indeed, recent studies have showed that COVID-19 bereavement yields much higher grief disorders than natural bereavement, similar to bereavement after an unnatural loss (e.g., suicide, homicide) (2). This intervention, if delivered by psychologists working at the hospital, could play both the function of closing the hospital care pathway (i.e., as hospital bereavement follow-up) and of eventually supporting families and preventing further psychological distress and grief disorders (by identifying individuals at risk and referring them to community-based services) (i.e., as psychological first aid). Further research should explore the potential impact of this one-shot intervention on families' psychological and psychopathological outcomes. The description of how the intervention was organized within the hospital care pathway and of its contents, components and structure can potentially provide indications to hospital organizations dealing with COVID-19 deaths about how to organize the support to bereaved families in the special circumstances of the pandemic and prevent later psychological issues. It can also provide detailed indications for psychologists delivering similar services about the specific challenges and actions that such an intervention may require to their profession.

## Data Availability Statement

The original contributions presented in the study are included in the article, further inquiries can be directed to the corresponding author.

## Author Contributions

LB and JM conceived the idea, collected the data, analyzed the data, and wrote the first draft of the manuscript. EV conceived the idea, revised the draft of the manuscript critically, contributed to the data interpretation, and gave the agreement for the final approval of the manuscript. The Early Bereavement Psychological Intervention working group performed the phone follow-up interventions, revised the draft of the manuscript critically, contributed to the data interpretation, and gave the agreement for the final approval of the manuscript. All authors contributed to the article and approved the submitted version.

## The Early Bereavement Psychological Intervention Working Group

Andrighi Elisa, Benassi Francesca, Bettini Laura, Biscardi Davide, Cassardo Claudio, Catanzaro Luigina, Colombi Francesca, Curatolo Antonella, Del Negro Silvia, Di Tucci Antonio, Fossati Ivan, Gazale Maria Fiorella, Lamberti Rosalba, Mariani Vera, Rabà Tamara, Raisi Simonetta, Rossi Paola, Troielli Walter, Valentini Tiziana. For all the member of the working group, the affiliation is “Unit of Clinical Psychology, ASST Santi Paolo and Carlo Hospitals, Milan, Italy,” except for Rossi Paola and Troielli Walter from whom the affiliation is “Addiction Unit, ASST Santi Paolo and Carlo Hospitals, Milan, Italy.”

## Conflict of Interest

The authors declare that the research was conducted in the absence of any commercial or financial relationships that could be construed as a potential conflict of interest.

## References

[1] Coronavirus Resource Centre COVID-19 Dashboard by the Center for Systems Science and Engineering (CSSE) at Johns Hopkins University. (2020). Retrieved from: https://coronavirus.jhu.edu/map.html (accessed october 28, 2020).

[2] Eisma MC Tamminga A Smid GE Boelen PA. Acute grief after deaths due to COVID-19, natural causes and unnatural causes: an empirical comparison. J Affect Disord. (2020) 278:54–6. 10.1016/j.jad.2020.09.049 32950843 PMC7487144

[3] Coombs M. A scoping review of family experience and need during end of life care in intensive care. Nursing Open. (2015) 2:24–35. 10.1002/nop2.14 27708798 PMC5047309

[4] Menichetti J Borghi L Cao di San Marco E Fossati I Vegni E Phone follow up to families of COVID-19 patients who died at the hospital: families' grief reactions and clinical psychologists' roles. Int. J. Psychol. (2021). 10.1002/IJOP.12742 PMC8013378 33511652

[5] Hudson P Remedios C Zordan R Thomas K Clifton D Crewdson M. Guidelines for the psychosocial and bereavement support of family caregivers of palliative care patients. J Palliat Med. (2012) 15:696–702. 10.1089/jpm.2011.0466 22385026 PMC3362953

[6] Virdun C Luckett T Lorenz K Davidson PM Phillips J. Dying in the hospital setting: a meta-synthesis identifying the elements of end-of-life care that patients and their families describe as being important. Palliat Med. (2017) 31:587–601. 10.1177/0269216316673547 27932631

[7] Gesi C Carmassi C Cerveri G Carpita B Cremone IM Dell'Osso L. Complicated grief: what to expect after the coronavirus pandemic. Front Psychiatry. (2020) 11:489. 10.3389/fpsyt.2020.00489 32574243 PMC7264152

[8] Buerhaus PI Auerbach DI Staiger DO. Older Clinicians and the Surge in Novel Coronavirus Disease 2019 (COVID-19). JAMA. (2020) 323:1777–8. 10.1001/jama.2020.4978 32227200

[9] Duan L Zhu G. Psychological interventions for people affected by the COVID-19 epidemic. Lancet Psychiatry. (2020) 7:300–2. 10.1016/S2215-0366(20)30073-0 32085840 PMC7128328

[10] Jiang X Deng L Zhu Y Ji H Tao L Liu L. Psychological crisis intervention during the outbreak period of new coronavirus pneumonia from experience in Shanghai. Psychiatry Res. (2020) 286:112903. 10.1016/j.psychres.2020.112903 32146245 PMC7112608

[11] Zhang J Wu W Zhao X Zhang W Recommended psychological crisis intervention response to the 2019 novel coronavirus pneumonia outbreak in China: a model of West China Hospital. Precision Clin Med. (2020) 3:3–8. 10.1093/pcmedi/pbaa006 PMC7107095 35960676

[12] Orrù G Ciacchini R Gemignani A Conversano C. Psychological intervention measures during the COVID-19 pandemic. Clin Neuropsychiatry. (2020) 17, 76–9. 10.36131/CN20200208 34908972 PMC8629089

[13] Leone D Borghi L Bonazza F Abrami MA Barcellini G Benlodi A. Psychological interventions in hospital during the first-wave of CoViD-19: an overview of the experiences of the Units of Clinical Psychology in Lombardy, Italy. [Interventi psicologici in ospedale ai tempi del COVID-19: una panoramica delle realtà proposte dalle Unità Operative di Psicologia (UOPSI) della Lombardia]. Recenti Prog Med. (2020) 111:593–601. 33078009 10.1701/3453.34419

[14] Wallace CL Wladkowski SP Gibson A White P. Grief During the COVID-19 Pandemic: considerations for palliative care providers. J Pain Symptom Manage. (2020) 60:e70–6. 10.1016/j.jpainsymman.2020.04.012 32298748 PMC7153515

[15] Selman LE Chao D Sowden R Marshall S Chamberlain C Koffman J. Bereavement Support on the Frontline of COVID-19: recommendations for hospital clinicians. J Pain Symptom Manage. (2020) 60:e81–6. 10.1016/j.jpainsymman.2020.04.024 32376262 PMC7196538

[16] Carr D Boerner K Moorman S. Bereavement in the time of coronavirus: unprecedented challenges demand novel interventions. J Aging Soc Policy. (2020) 32:425–31. 10.1080/08959420.2020.1764320 32419667

[17] Borghi L Vegni EAM Raisi S Cardani E de Stasio M Leone D The psychological consultation in hospital setting: a model of intervention through the clinical practice. [La visita e parere psicologico in ambito ospedaliero un modello di intervento attraverso la pratica clinica]. Psicol della Salute. (2020) 20:55–67. 10.3280/PDS2020-001005

[18] Mason TM Tofthagen CS Buck HG. Complicated grief: risk factors, protective factors, and interventions. J Soc Work End Life Palliat Care. (2020) 16:151–74. 10.1080/15524256.2020.1745726 32233740

[19] Stroebe M Schut H Stroebe W. Health outcomes of bereavement. Lancet. (2007) 370:1960–73. 10.1016/S0140-6736(07)61816-9 18068517

[20] Neimeyer RA Burke LA Mackay MM van Dyke Stringer JG Grief therapy and the reconstruction of meaning: from principles to practice. J Contemp Psychother. (2010) 40:73–83. 10.1007/s10879-009-9135-3

[21] Agnew A Manktelow R Haynes T Jones L Bereavement assessment practice in hospice settings: challenges for palliative care social workers. Br J Soc Work. (2011) 41:111–30. 10.1093/bjsw/bcq055

[22] Cunha S Soares-Oliveira M Pereira N. Early psychological intervention in prehospital emergency care systems. J Emerg Med. (2009) 36:404–6. 10.1016/j.jemermed.2007.10.031 18385002

